# Pathogenic Role of Circulating Citrullinated Antigens and Anti-Cyclic Monoclonal Citrullinated Peptide Antibodies in Rheumatoid Arthritis

**DOI:** 10.3389/fimmu.2021.692242

**Published:** 2021-06-30

**Authors:** Pureun Won, Youngkyun Kim, Hyerin Jung, Yeri Alice Rim, Dong Hyun Sohn, William H. Robinson, Su-Jin Moon, Ji Hyeon Ju

**Affiliations:** ^1^ Clinical Immunology and Stem Cell Laboratory (CiSTEM) Laboratory, Catholic Induced Pluripotent Stem Cell (iPSC) Research Center, College of Medicine, The Catholic University of Korea, Seoul, South Korea; ^2^ Department of Biomedicine & Health Science, Seoul St. Mary’s Hospital, College of Medicine, The Catholic University of Korea, Seoul, South Korea; ^3^ Oncology and Immunology Research Center, Lucky-Goldstar (LG) Chem., LG Science Park, Seoul, South Korea; ^4^ Department of Veterans Affairs (VA) Palo Alto Healthcare System, Palo Alto, CA, United States; ^5^ Division of Immunology and Rheumatology, Stanford University School of Medicine, Stanford, CA, United States; ^6^ Department of Microbiology and Immunology, Pusan National University School of Medicine, Yangsan, South Korea; ^7^ Division of Rheumatology, Department of Internal Medicine, Uijeongbu St. Mary’s Hospital, College of Medicine, The Catholic University of Korea, Seoul, South Korea; ^8^ Division of Rheumatology, Department of Internal Medicine, Seoul St. Mary’s Hospital, College of Medicine, The Catholic University of Korea, Seoul, South Korea

**Keywords:** rheumatoid arthritis, citrullination, cyclic citrullinated peptide, monoclonal antibody, diagnosis

## Abstract

We examined whether it is possible to directly detect citrullinated antigens in the serum of rheumatoid arthritis (RA) patients using a monoclonal antibody (mAb) designed to be specific for citrullinated peptides. In order to confirm the potential of the mAb as a direct arthritis-inducing substance through experimental model of RA, a monoclonal antibody (mAb) 12G1 was generated using by immunization of mice with a challenging cyclic citrullinated peptide. Immunohistochemical analysis of RA-affected synovial tissue showed that our mAb 12G1 could indeed detect citrullinated proteins in target tissues. Subsequently, serum levels of citrullinated type II collagen and filaggrin were measured in healthy volunteers, patients with RA, ankylosing spondylitis (AS), and systemic lupus erythematosus (SLE) using a 12G1-based sandwich ELISA. This showed that citrullinated filaggrin showed 78.9% sensitivity and 85.9% specificity for RA diagnosis with a cutoff optical density (OD) value of 1.013, comparable with the results from a second-generation anti-citrullinated protein antibody (ACPA) test. Circulating citrullinated collagen and filaggrin were detected even in sera of RA patients who were negative for both rheumatoid factor (RF) and ACPA. ELISA results also showed that RF and ACPA titers showed significantly positive correlation with both citrullinated collagen and filaggrin OD values in sera of RA patients. 12G1 challenging aggravated the severity of murine arthritis. In summary, mAb 12G1 can directly detect citrullinated proteins in RA target tissue and in sera of RA patients and 12G1 showed direct arthritogenic potential *in vivo*. This, 12G1 might be useful for diagnosis of RA including seronegative RA and may help to elucidate the pathophysiological role of citrullination in RA.

## Introduction

Rheumatoid arthritis (RA) is a chronic, systemic inflammatory disease characterized by inflammation of the synovial membrane lining the joints and progressive joint damage. Although the pathogenesis of RA remains uncertain, it is increasingly clear that altered cellular immune responses play a role (1). The presence of autoreactive T cells and autoantibodies are the main characteristics of RA, and both are detectable in the earliest stages of the disease. Autoantibodies, including rheumatoid factor (RF), can be detected in serum and synovial fluid (SF) samples from RA patients.

Citrullination is a post-translational modification performed by the enzyme peptidyl-arginine deiminase (PAD), which converts the amino acid arginine into the noncoded amino acid citrulline. This modification can be detected in the inflamed joints of mice and in RA-affected synovial tissue in humans (2–6). Proteins harboring citrullinated epitopes are the dominant antigens recognized by autoantibodies in serum from RA patients (7, 8). For example, filaggrin contains many arginine residues, and citrullinated anti-filaggrin antibodies purified from RA sera can recognize (pro)filaggrins only when the filaggrins are citrullinated.

The presence of anti-citrullinated protein antibodies (ACPAs) is a hallmark of RA (9). After the presence in the blood of RA patients and its clinical significance of ACPA was revealed, the pathophysiological significance of ACPA or citrullination has been shown through numerous studies (10, 11). As the generation of citrullinated antigens and resulting ACPA is directly involved in RA pathogenesis regarding the development or progression of the disease, studies have been conducted on the expression of ACPA-producing B cells and its immunological characters (12, 13). Recent studies showed a mechanism of epitope spreading of ACPA response against citrullinated antigens through ACPA plasmablast repertoires (14, 15). Indeed, ACPA derived from RA patients can specifically recognize citrulline residue largely independent of the peptide epitope sequence (16). One recent paper by Tilvawala et al. showed that RA-associated citrullinated proteins in sera, synovial fluids, and synovial tissues samples by proteomic analysis (17). They identified more than 150 novel citrullinated proteins and validated the role of citrullination on target proteins identified in their study (17).

Based on the previous reports that citrullinated antigens are present in joints, synovial fluid and synovial tissues of RA patients, it is possible that soluble citrullinated autoantigens can be detected in the serum or SF of RA patients using a sandwich enzyme-linked immunosorbent assay (ELISA). Some previous studies have verified a method for detecting citrullination. Senshu et al. developed a method for detecting citrullinated proteins using polyvinylidene difluoride membranes (18). A decade after that report, an IgM monoclonal antibody (mAb), designated F95, was developed using hybridoma technology (19). F95 staining revealed the increased presence of intracellular citrullinated proteins in RA-affected synovial tissue (53%) compared with control tissue (5%), but the extracellular staining pattern from F95 was not RA-specific. F95 was generated against a deca-citrullinated peptide comprising 10 citrulline residues and a carrier protein, and it was inconclusive whether F95 could distinguish citrulline residues from arginine residues in the target proteins. No study has reported the detection of intact citrullinated peptides with an artificially designed antibody specific for circular citrullinated peptides (CCPs).

The goal of the present study was to generate a mAb that recognizes diverse citrullinated proteins and to confirm its ability to detect citrullinated autoantigens directly in RA samples. We generated mAb, named 12G1, to detect citrullinated proteins directly in RA-affected synovial tissue. A sandwich ELISA was developed based on 12G1-detected citrullinated autoantigens in sera from RA patients. The levels of citrullinated collagen, and filaggrin were significantly higher in RA sera than in sera of healthy control, ankylosing spondylitis (AS), or systemic lupus erythematosus (SLE) patients. Interestingly, higher citrullinated filaggrin levels were detected in RA patients who had neither RF nor ACPA (seronegative) compared with controls. The optical density (OD) values of citrullinated collagens and filaggrin in RA patients correlated positively with both the rheumatoid factor (RF) and ACPA titers. 12G1 showed arthritogenic potential in a murine model of inflammatory arthritis in our present study.

## Materials and Methods

### Mice

Female BALB/c mice (6 weeks of age) and female DBA1/J mice (5 weeks of age) were purchased from OrientBio (Seongnam, Korea) and housed in specific pathogen–free conditions. All procedures involving animals were in accordance with the Laboratory Animals Welfare Act, the Guide for the Care and Use of Laboratory Animals, and the Guidelines and Policies for Rodent Experimentation provided by the Institutional Animal Care and Use Committee of the School of Medicine at the Catholic University of Korea. This study protocol was approved by the Institutional Review Board of the Catholic University of Korea (CUMC-2011-0062-03 and CUMC-2018-0338-01).

### Immunization of Mice and Preparation of the mAb

A CCP (HQCHQEST**X**GRSRGRCGRSGS; X = citrulline) and a non-citrullinated peptide (NCP: HQCHQEST**R**GRSRGRCGRSGS) were synthesized. The synthetic CCP was mixed with complete Freund’s adjuvant and used to immunize four 6-week-old female BALB/c mice *via* injection into the abdominal cavity. For boosting, the mice were injected with CCPs diluted in phosphate-buffered saline (PBS) 4 and 8 weeks after the first immunization. Three days later, B cells were isolated from the mouse with the highest binding reactivity against the CCPs in serum, as measured by ELISA (details are provided in the method described below) and fused with myeloma cells (Sp2/0-Ag14) using polyethylene glycol (Roche, Basel, Switzerland).

The fused cells were then cultured in hypoxanthine–aminopterin–thymidine culture medium (Sigma, St. Louis, MO, USA). Cells showing a positive signal in the ELISA were transferred to a 24-well plate. After individual cells were placed into separate wells in 96-well plates, the cells were cultured for 7–10 days in hypoxanthine and thymidine culture medium (Gibco/Thermo Fisher Scientific, Waltham, MA, USA) in a 5% CO2 incubator at 37°C. Hybridoma cells were screened by ELISA, and the cloning process was repeated until the final antibody-secreting clone was selected.

### ELISA for Antibody Screening

CCPs and NCPs (negative control) were diluted to 5μg/ml in coating buffer, and 50μl were coated in separate wells of an ELISA plate either overnight at 4°C or for 2 hours at 37°C. The plates were blocked with 2% skimmed milk in Tris-buffered saline with Tween 20 at 37°C for 1 hour. Serum from immunized mice or the supernatant from the hybridoma cells was added to the wells, and the plates were incubated for 2 hours at RT. The plates were washed, and 50μl of horseradish peroxidase (HRP)-conjugated goat anti-rabbit IgG was added to each well for 1 hour at RT. Finally, 50 μl of chromogenic substrate (SurModics, Eden Prairie, MN, USA) was added to each well, and the plates were incubated for 30 minutes, after which 50μl of stop solution (1N H2SO4) was added. The absorbance was read at 450 nm in a VERSAmax ELISA reader. Serum samples were collected from mice 4 weeks after their primary immunization and tested as just described.

### Total RNA Extraction and Synthesize cDNA of Antibody Variable Regions

The monoclonal antibody-producing hybridoma cells were generated as described above (**2.2**), and total RNA was extracted using the Easy-Blue total RNA extraction kit (Intron Biotechnology, Sungnam, Korea) according to the manufacturer’s instructions. In addition, oligo dT primer with reverse transcriptase (Promega, Madison, WI, USA) was used to reverse transcribe the extracted RNA into cDNA, and heavy and light chain genes of our synthetic monoclonal antibody were amplified from cDNA using Ex-Taq DNA polymerase (Takara Bio, Shiga, Japan) with specific primers described (20) and cloned whole amino acids and nucleotides for the antibody using as described (20).

### *In Vitro* Citrullination

Human type II collagen (Creative Biomart, Shirley, NY, USA), fibronectin (Sigma-Aldrich/Thermo Fisher Scientific), and filaggrin (Biomatik, Cambridge, Ontario, Canada) were subjected to *in vitro* citrullination with PAD derived from rabbit skeletal muscle (Sigma-Aldrich) at 5 units/mg of protein for 3 hours at 48°C in citrullination buffer containing 100 mM Tris-HCl (pH 7.4), 10 mM CaCl2, and 5 mM dithioerythritol.

### Detection of *In Vitro* Citrullinated Proteins by Artificial Anti-Citrullinated Protein Antibody

In the ELISA, 96-well plates were coated with 10 μg/ml of *in vitro* citrullinated and non-citrullinated proteins overnight at 4°C. Plates were washed and blocked with 1% BSA for 1 hour at RT, washed again, and incubated with 20 μg/ml of the mAb 12G1 for 2 hours at RT followed by incubation with HRP-conjugated anti-mouse IgG for 1 hour at RT. The OD at 450 nm was measured after treatment with 3,3’,5,5’-Tetramethylbenzidine (TMB substrates; ebioscience/Thermo Fisher Scientific) and stop solution (1N H_2_SO_4_).

### Western Blotting and Isotyping

The synthetic peptides, CCPs and NCPs, are too small to be detected by Western blotting, so we conjugated them to BSA. Citrullinated and non-citrullinated proteins were loaded onto SDS-PAGE gels. After electrophoresis, the proteins were transferred to nitrocellulose membranes, which were then incubated with the antibody of interest in blocking buffer (5% skimmed milk in PBS containing 0.05% Tween 20 (PBS-T)) for 1 hour at RT or 12–14 hours at 4°C. After washing with blocking buffer, the bound antibodies were detected by incubation with HRP-conjugated secondary antibodies and visualized by chemiluminescence. The subtype of mAb 12G1 was analyzed using the mouse monoclonal antibody isotyping kit (Invitrogen/Thermo Fisher Scientific, Waltham, MA, USA) following the manufacturer’s instruction.

### Immunohistochemistry

Synovial tissues from RA patients, OA patients, and healthy donors were embedded in paraffin, sectioned, mounted on slides, and baked at 60°C for 60 minutes. The sections were then deparaffinized and rehydrated through a series of graded alcohols, ending with a rinse in tap water. The slides were incubated with 3% hydrogen peroxide for 15 minutes to block endogenous peroxidase. Next, the slides were washed with tap water and blocked with 10% normal horse serum plus 1% BSA in PBS. After washing with PBS-T, the slides were incubated with the mAb 12G1 (1:50) overnight at 4°C. A biotinylated horse anti-mouse IgG secondary antibody (1:200) was applied for 40 minutes at RT, followed by the R.T.U. VECTASTAIN^®^ Elite ABC reagent for 10 minutes. Tissue staining was visualized after incubation with the DAB substrate chromogen solution (Vector Laboratories, Burlingame, CA, USA) for 1 minute 30 seconds. Slides were counterstained with hematoxylin for 1 minute, dehydrated, and mounted.

For mouse tissue, joints were embedded in paraffin, sectioned, deparaffinized in xylene, and hydrated using serially diluted ethanol ending with a rinse in tap water. To block endogenous peroxidase, the sections were incubated in 3% H_2_O_2_ diluted in PBS for 15 min and washed with tap water. The slides were incubated with Mouse on Mouse (M.O.M.) IgG blocking agent from a M.O.M. Basic Kit for 30 min to block nonspecific antibody binding. The 12G1 mAb (1:100) was diluted in M.O.M. diluent buffer and incubated overnight at 4°C. A biotinylated anti-mouse Ig reagent was applied for 10 mins at RT followed by R.T.U. VECTASTAIN^®^ Elite ABC reagent for 10 mins. The sections were then incubated with DAB solution for 1 min. Slides were counterstained with hematoxylin for 30 secs, dehydrated, and mounted.

For all cases, the isotype (negative) control was a mouse IgG2 mAb (Cell Signaling Technology, Danvers, MA, USA) and the slides were visualized using Leica DM500B imaging system (Leica Microsystems Ltd., Wetzlar, German).

### Sandwich ELISA for the Detection of Citrullinated Proteins in Human Serum

Briefly, 96-well microtiter plates were coated with polyclonal anti-collagen (Abcam, Cambridge, UK), anti-filaggrin (Santa Cruz Biotechnology, Dallas, Texas, USA), or anti-fibronectin (Abcam) antibodies (1:100 dilution) in 100μl of coating buffer (eBioscience) and incubated overnight at 4°C. The plates were washed seven times with PBS-T and incubated with assay buffer (eBioscience) for 1 h at room temperature (RT). The plates were again washed seven times with PBS-T. Serum samples (1:10 diluted in PBS) were added to each well in triplicate, and the plates were incubated for 2 h at RT. The plates were washed seven times with PBS-T, and then the 12G1 mAb (1:100 diluted in assay buffer) was added, and the plates were incubated for 2 h at RT. After incubation, the plates were washed seven times in PBS-T, 50µl of HRP-conjugated anti-mouse IgG antibody (1:3000, Amersham Pharmacia Biotech, Amersham, Buckinghamshire, UK) was added, and the plates were incubated for a further 1 hour at 37 °C. Finally, the plates were washed nine times, and the bound antibodies were visualized by adding substrate solution (eBioscience). Reactions were stopped after 15 mins by adding 50µl of stop solution (eBioscience). The colorimetric reaction was measured at 450 nm on a VersaMax ELISA reader.

### Human Subjects

Synovial tissue samples were obtained from RA patients and OA patients at the time of total knee replacement surgery. Serum samples were obtained from 148 RA patients (106 women and 42 men), 57 AS patients (18 women, and 39 men), and 60 SLE patients (1 man, and 59 women) who visited the Outpatient Department of the Division of Rheumatology at Seoul St. Mary’s Hospital at the Catholic University of Korea between May 2015 and August 2018. All RA patients who met both the American College of Rheumatology (ACR) criteria for RA (21) and the 2010 ACR/European League Against Rheumatism criteria (22) were included in the study. AS and SLE patients who met ASAS classification criteria (23) and 2012 SLICC SLE criteria (24), respectively. Seventy-one healthy volunteers (62 women and 9 men) were included as controls. Elderly onset RA (EORA) was defined as RA onset age at ≥60 years, and young onset RA (YORA) was defined as RA symptoms developing between the ages of 18 and 59 years. Early RA (ERA) was defined as a disease duration of <6 months.

Serum samples were stored at –80°C until analysis. This study was approved by the Institutional Review Board of Seoul St. Mary’s Hospital and was performed in accordance with the Declaration of Helsinki. All patients provided written informed consent.

### Clinical Data and Measurement of Inflammatory Markers

Data about the age, sex, disease duration, Erythrocyte sedimentation rate (ESR), C-reactive protein (CRP) level, ACPA level, and Rheumatoid factor (RF) titer were obtained for each patient. The RF titer was measured using a particle-enhanced immunoturbidimetric assay, and a level >15 U/ml was considered positive. The ACPA titer was measured in a chemiluminescent microparticle immunoassay (ARCHITECT anti-cyclic citrullinated peptide assay), which is a second-generation test for ACPAs; a level higher than the cutoff value of 5 U/ml was considered positive (as suggested by the manufacturer). ESR was measured using the Westergren method. CRP was measured using an immunonephelometric method, and values >5 mg/l were considered positive.

### Induction of Mouse Models

The mice were organized into 4 groups of 5 mice each: CIA (collagen induced arthritis) as the normal control; vehicle as the negative control; 12G1; and 12G1 with lipopolysaccharide (LPS). To induce CIA, 2mg/ml of bovine type II collagen (CII; Chondrex, Redmond, WA, USA) emulsified with 2mg/ml of complete Freund’s adjuvant (Chondrex) was given intradermally to 6-week-old DBA1/J mice. After 21 days, the same concentration of CII emulsified with incomplete Freund’s adjuvant (IFA; Chondrex) was injected into the CIA mice. To induce arthritis using the 12G1 mAb, 12G1 mAb diluted with PBS (1.5mg per mouse) was injected instead of the CII and IFA mixture. After 7 days, either the same concentration of 12G1 mAb was injected again or LPS was given at 50µg/µl. The same concentration of 12G1 mAb was combined with CCP chelating beads (Santa Cruz Biotechnology) for 4 h at 4°C, which was followed by centrifugation (250 g, 30 s); the resulting supernatants were collected and injected into the mice as vehicle.

The development of arthritis was monitored and scored in a blinded manner. Disease severity was scored three times a week. The severity of arthritis in each front and hind paw was scored from 0 to 4 (0, normal; 1, mild swelling confined to the tarsals; 2, swelling of two or more toes or joints, or increased swelling; 3, moderate swelling extending from the ankle to the metatarsal joints; and 4, severe swelling encompassing the ankle, foot, and digits). A representative arthritis score was determined by summing the scores of all four paws (25).

### Histological Analysis

Joint tissues from hind paws were fixed in 4% paraformaldehyde, decalcified in 10% EDTA bone decalcifying solution, and embedded in paraffin. Paraffin sections of 4μm thickness were prepared and stained with H&E, safranin O, and toluidine blue.

The inflammation and joint destruction scores were measured microscopically using the procedure of Huckel et al. by three individual researchers in a blinded manner (26). The inflammation score was measured using the severity of infiltration and pannus formation. The destruction score was measured based on cartilage and bone destruction (27, 28).

### Flow Cytometry Analysis

The spleen samples were dissociated into single-cell suspensions, and 2×10^6^ cells were transferred to round-bottom polystyrene tubes (BD Biosciences, CA, USA). After washing with PBS supplemented with 2% FBS, mouse spleen cells were stained with a rat anti-mouse CD4 (2μg/mL) antibody conjugated with allophycocyanin (APC) (eBioscience, CA, USA). The cells were then permeabilized using Flow Cytometry Fixation and Permeabilization buffer (eBioscience) and stained with an anti-human/mouse RORγt (2μg/mL) antibody conjugated with phycoerythrin (eBioscience). The flow cytometry analysis was performed using a BD LSR Fortessa cell analyzer (BD Biosciences). To analyze the data, FlowJo V10 Single Cell Analysis Software was used (TreeStar Inc., OR, USA).

### Statistical Analysis

Data were analyzed using SPSS software version 18.0 (SPSS Inc., Chicago, IL, USA) and are expressed as the mean ± SEM or number (%). The mean values were compared between multiple groups using one-way analysis of variance followed by Tukey’s test. The Shapiro–Wilk test and the Levene test were used to assess Gaussian distributions and the equality of variance, respectively. Qualitative data were compared using the chi-squared test. ROC curves were constructed by calculating the specificity and sensitivity of each OD value at different cutoff points, and the area under the curve was computed. Sensitivity was defined as the percentage of subjects with disease (RA or seronegative RA) who showed a positive result for citrullinated antigens in the ELISA. Specificity was defined as the percentage of subjects without RA who had a negative ELISA result. The PPV was calculated as the number of true positives (positive ELISA result plus confirmed RA) divided by the number of subjects with a positive ELISA result. The NPV was calculated as the number of true negatives (negative ELISA result and no RA) divided by the number of subjects with a negative ELISA result. All *P* values were two-tailed. *P* values <0.05 were considered significant.

## Results

### Generation of a mAb Against CCPs

**Figure 1:**
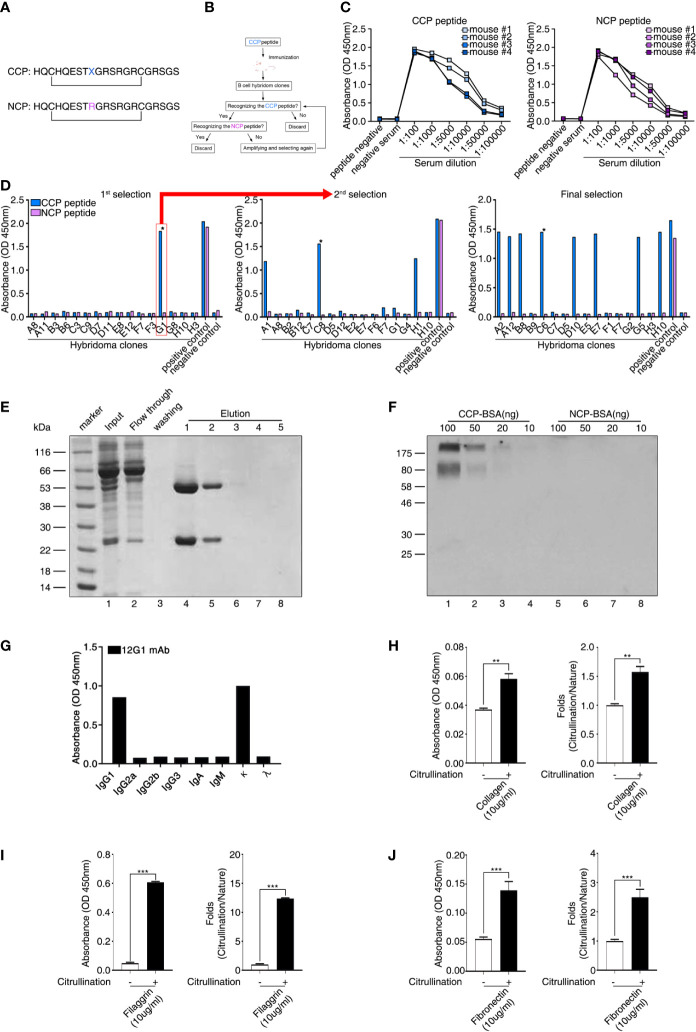
Generation of a cyclic citrullinated peptide (CCP)–specific monoclonal antibody (mAb). **(A)** Sequences of the two synthetic peptides used as antigens. Both peptides shared the same sequence, except that the CCP was generated by replacing an arginine residue with a citrulline residue (X = citrulline). The bar indicates a disulfide bond between the two cysteines, which generates a circular peptide. **(B)** Schematic diagram showing the strategy for selecting hybridoma clones. Hybridoma clones that secreted antibodies specific to CCP were obtained by repeated selection. **(C)** Selection of mice showing seroreactivity against the CCP. After the first injection of the CCP, the binding reactivity to the CCP and the cyclic arginine peptide (NCP) was examined using ELISA (serum samples diluted 1:100 to 1:100000). **(D)** ELISA of fused hybridoma clones that secreted mAbs to the CCP. After fusion, hybridoma clones secreting anti-CCP antibodies specific for the CCP but not the NCP were selected. Clones reactive to the CCP were isolated and evaluated repeatedly until a single clone secreting high-reactivity anti-CCP antibodies was selected. **(E)** The 12G1 mAb was purified from the hybridoma culture supernatant using a Protein G agarose column. After the supernatant (input) was loaded onto the column, bound 12G1 mAb was eluted in the elution buffer (elution). The purified antibody was confirmed by SDS-PAGE and Coomassie brilliant blue staining. **(F)** The specificity of the 12G1 mAb for the CCP was examined by Western blotting. CCP–BSA and NCP–BSA conjugates were run in an SDS-PAGE gel and then immunoblotted with the 12G1 mAb. **(G)** mAb 12G1 immunoglobulin isotyping assay (absorbance of positive response ≥ 0.2). **(H–J)** The 12G1 mAb-based ELISA system effectively detected citrullinated collagen **(H)**, citrullinated filaggrin **(I)**, and citrullinated fibronectin **(J)**. *P < 0.05; **P < 0.01; ***P < 0.001.

To identify the presence of circulating autoantigens that could induce the production of ACPAs, we generated a mAb specific for citrullinated antigens. First, we synthesized a CCP and an NCP control peptide (cyclic arginine peptide) in which citrulline was replaced with arginine (**Figure 1A**). This synthetic peptide included citrulline in a particular region of the filaggrin subunit that is cyclized *via* a disulfide bond and forms a structure that favors recognition by RA autoantibodies (29). We then used hybridoma technology to generate high-quality mAbs with reactivity for the CCP but not the NCP (**Figure 1B**). Four mice were immunized with the CCP, and we examined the reactivity of their serum antibodies to the CCP and NCP (**Figure 1C**). We selected mouse number 2 because its serum samples showed strong binding reactivity to the CCP and weak binding to the control NCP (**Figure 1C**). Hybridoma clones were generated by fusing a myeloma cell line with B cells derived from the selected CCP-immunized mouse. We then performed an ELISA to identify antibody-secreting clones that produced anti-CCP antibodies (**Figure 1D** and **Supplementary Figure 1**). This process was repeated until we isolated a single clone (designated 12G1) that secreted a mAb with high reactivity to the CCP but not to the control peptide (asterisk in **Figure 1D**, final selection). The binding affinity of 12G1 was confirmed (**Supplementary Figure 2A**). The isotype of the artificial anti-citrullinated protein antibody (12G1) was IgG1, and the V regions of the light and heavy chains of 12G1 were sequenced (**Supplementary Figure 2B**).

### 12G1 mAb Is Specific for Citrulline

To confirm that the selected hybridoma cell line could secrete intact 12G1, we purified the antibody from the culture supernatant using antibody-binding beads (**Figure 1E**). Subsequent electrophoresis in SDS-PAGE gels confirmed that the purified 12G1 had a normal antibody structure; i.e., a heavy chain and a light chain (**Figure 1E**, lanes 4, 5). In addition, to ensure that the 12G1 recognized citrulline, we performed an immunoblot assay using the CCP and NCP as target antigens (**Figure 1F**). Because the CCP and NCP were too small to detect using SDS-PAGE gels, we conjugated them to bovine serum albumin (BSA). The 12G1 mAb detected the CCP specifically and in a dose-dependent manner (**Figure 1F**, lanes 1-4). As expected, the 12G1 mAb did not react with the NCP (**Figure 1F**, lanes 5-8). These data clearly demonstrate that the 12G1 mAb was specific for the citrullinated peptide. Immunoglobulin isotyping assay showed that the heavy chain and light chain of 12G1 were identified as IgG1 and kappa chain, respectively (**Figure 1G**). Next, we examined whether the 12G1 mAb could be available in the sandwich ELISA system. If so, we reasoned that our aACPA would have clinical use in the diagnosis of diverse autoimmune and autoinflammatory diseases involving citrullination. *In vitro* citrullination experiments were performed using PAD. Six naïve target antigens (human type II collagen, filaggrin, enolase, BiP, vimentin, and fibronectin) and their corresponding citrullinated antigens were tested to determine whether the aACPA could detect the citrullinated forms of the target antigens. Among them, citrullinated-collagen, -filaggrin and -fibronectin could be detected by the 12G1 in the sandwich ELISA system (**Figures 1H–J**). These results confirm both that our mAb 12G1 is specific for citrullinated antigens and that it can be used in a sandwich ELISA system.

### 12G1 Detects Citrullinated Autoantigens in Synovial Tissues From RA Patients

**Figure 2:**
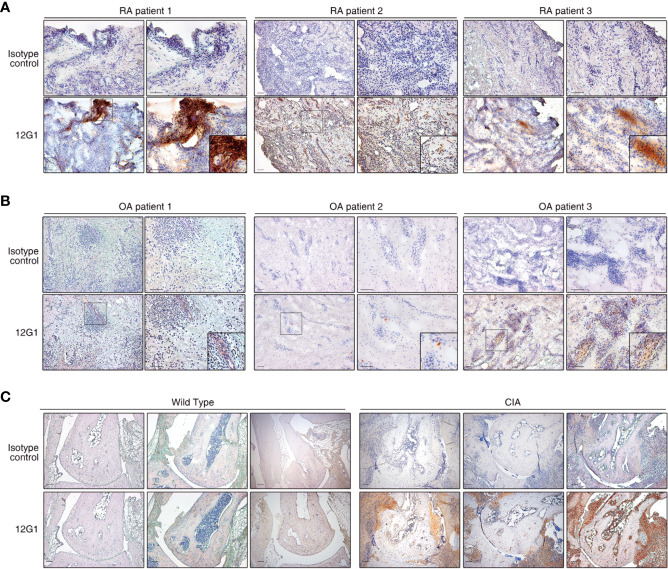
The 12G1 mAb is specific for citrullinated proteins in RA synovial tissue. Representative images showing immunohistochemical (IHC) staining of citrullinated proteins in RA and OA. **(A)** The IHC analysis was performed using the 12G1 mAb in joint synovium tissue specimens from three RA patients (RA patients 1-3). To ascertain whether any antigens could be detected by the 12G1 mAb in noninflammatory synovial tissues, synovial tissues from three OA patients **(B)** were stained with 12G1 mAb at the same time. An isotype control antibody was used as a negative control. **(C)** A joint with collagen-induced arthritis (CIA) was stained with the 12G1 mAb. The black box shows the stained synovial tissue at a higher magnification. The magnification is indicated for each image in this figure. All scale bars represent 100 µm.

Because citrullinated proteins can be detected in inflamed RA synovial tissue, we next examined whether 12G1 could specifically detect such proteins in synovial tissues isolated from RA patients. Immunohistochemical staining of synovial tissue showed that 12G1 could detect citrullinated proteins in RA tissues, as we expected (**Figure 2A**). Control staining of the same tissues with an irrelevant isotype-matched primary antibody gave negative results (**Figure 2A**, upper panels). 12G1 recognized citrullinated proteins in synovial tissue from three different RA patients. To confirm that 12G1 was specific for citrullination, we also examined synovial tissues isolated from three patients with OA (**Figure 2B**). No positive staining was detected in the OA tissues. These findings suggest that the citrullinated autoantigens detected by 12G1 exist in RA-affected synovial tissue but not in OA-affected tissues. Taken together, the data obtained thus far suggest that the 12G1 could detect citrullinated proteins in RA synovial tissue and that it has potential for RA diagnosis. Next, 12G1 was used for tissue staining to determine whether the joints of CIA mice had citrullinated proteins. The results showed that CIA-affected joints contained citrullinated proteins in synovium, but wild-type mice did not (**Figure 2C**).

### Circulating Citrullinated Antigens Are Detected by 12G1 in Serum From RA Patients

**Figure 3:**
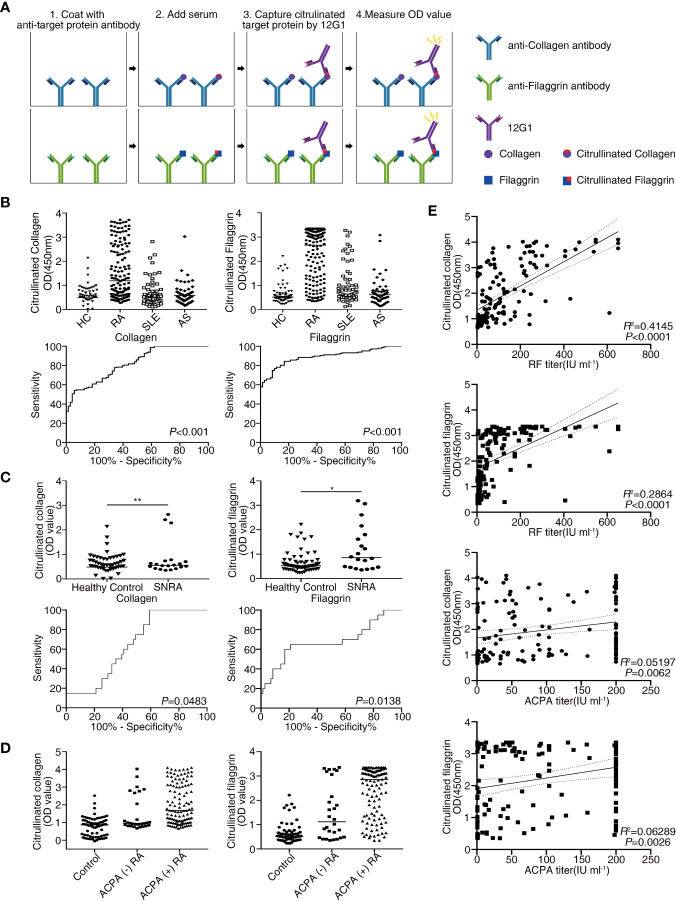
Receiver operating characteristic (ROC) curve analysis comparing the sensitivity and specificity of the ELISA for measuring the levels of citrullinated collagen, and citrullinated filaggrin in patient serum. Anti-collagen, and anti-filaggrin were coated onto plates to capture the candidate antigens. Citrullination was detected by the 12G1. **(A)** Schematic diagram showing the method for the sandwich ELISA using an anti-target protein antibody and 12G1. **(B)** Circulating citrullinated antigens in serum samples from RA (n = 148), SLE (n = 60), AS (n = 57) patients and control subjects (n = 71) (upper row) and the ROC curve for each target antigen (lower row). **(C)** Circulating citrullinated antigens in the sera of seronegative RA (n = 20) and control subjects (n = 71) (upper row) and the ROC curve for each target antigen (lower row). Specificity (true negative rate [the percentage of RA patients correctly predicted]) is plotted on the x-axis, and sensitivity (the percentage of RA patients correctly predicted) is plotted on the y-axis. Calculations were based on the predicted probability of each subject being above the cutoff OD values. *P* values were calculated using the Mann-Whitney *U* test. *P < 0.05. **(D)** OD values of three circulating citrullinated autoantigens for the control, ACPA-negative RA, and ACPA-positive RA subjects. **(E)** Correlations between circulating citrullinated collagen and filaggrin OD values and RF titers (n =146, r =0.644 for collagen, r = 0.535 for filaggrin, *P* < 0.0001), and ACPA titers (n = 143, r = 0.228 for collagen, r = 0.251 for filaggrin, *P* < 0.01) in RA patients (Pearson’s correlation coefficient). **P < 0.01.

Citrullination of target epitopes occurs in rheumatoid joints (6). Previous studies have shown that citrullinated proteins such as collagen, filaggrin, and fibronectin are implicated in the pathogenesis of RA (7, 30, 31). We developed a sandwich ELISA based on 12G1 to detect only the citrullinated proteins in sera from RA patients (Materials and Methods and **Figure 3A**). Serum samples were obtained from 148 RA patients and 71 age- and sex-matched healthy controls. To determine whether the presence of circulating citrullinated antigens in blood was RA-specific observation, ELISA was performed simultaneously in the sera of AS and SLE patients (**Figure 3B**).

The baseline clinical and laboratory characteristics of the patients and controls are presented in **Table 1**. Most of the RA patients were seropositive for either RF (71%) or ACPA (79%). Fifty-two of the RA patients (35.1%) had ERA (disease duration <6 months) and 43 (26.1%) had EORA (age of RA onset ≥60 years). Among RA patients taking medications (n = 106), sixty-six (62%) RA patients had received prednisolone (mean dose <5 mg/day).

**Table 1:** Clinical and laboratory findings in healthy controls and RA patients.

	Healthy controls	RA^a^	*P* value
	n = 71	n = 148	
Age (years)	55.5 ± 7.4	57.2 ± 12.2	NS
Women	62 (83.7%)	106 (71.6%)	NS
Disease duration (years)		3.5 ± 4.5	
RA onset age (years)		54.2 ± 13.1	
ESR^b^ (mm/h)		37.7 ± 27.9	
CRP^c^ (mg/dl)		0.93 ± 1.82	
RF^d^-positivity	0 (0)	105 (71%)	
ACPA^e^ positivity	0 (0)	118 (79%)	
Seropositive RA		128 (86%)	
ERA^f^ (<6 months)		52 (35.1%)	
EORA^g^ (onset age ≥60 years)		43 (29.1%)	
Tx^h^ naïve		42 (28.4%)	
**Current medication**			
Methotrexate		87 (82%)	
Leflunomide		31 (29%)	
Tacrolimus		11 (10%)	
Sulfasalazine		13 (12%)	
Hydroxychloroquine		29 (27%)	
Biologics use		5 (4.7%)	
Prednisolone use (%)		66 (65%)	
Mean prednisolone dose (mg/day) for the past month		2.7 ± 2.7	

Data are expressed as the mean ± SD or number (percentage).

a RA, rheumatoid arthritis; ^b^ESR, erythrocyte sedimentation rate; ^c^CRP, C-reactive protein; ^d^RF, rheumatoid factor; ^e^ACPA, anti-cyclic citrullinated peptide antibody; ^f^ERA, early rheumatoid arthritis; ^g^EORA, elderly onset rheumatoid arthritis; ^h^Tx, treatment. NS means No significant.

The OD values from the ELISA were analyzed for all the subjects enrolled in our study. The mean OD values for citrullinated collagen, and filaggrin were significantly higher in RA patients than in controls, SLE and AS patients (**Figure 3B**, upper panel). On the contrary, OD values of fibronectin in sera of RA patients did not increase compared to HC, SLE and AS patients (data not shown). We completed a receiver-operating characteristic (ROC) curve analysis and used the areas under the curve to calculate the diagnostic accuracy of these two target antigens (**Figure 3B**, lower panel). The mean OD values of citrullinated collagen and filaggrin in healthy controls and RA sera are shown in **Supplementary Table 1**. ROC curve analysis and the AUCs were used to calculate the diagnostic accuracy of target antigens (**Figures 3B, C** and **Supplementary Table 2**). The sensitivity, specificity, likelihood ratio, positive predictive value (PPV), negative predictive value (NPV), and accuracy of RA diagnosis according to the presence of citrullinated filaggrin were 78.9%, 85.9%, 5.6, 92%, 66.3%, and 80.8%, respectively, when using an OD cut-off value of 1.013. Changing the OD cut-off value changed the sensitivity, specificity, PPV, NPV, and accuracy (**Table 2**). Interference by RF that might have caused additional false-positive activity was not present in our 12G1-based ELISA system (data not shown). Taken together, these results suggest that citrullinated collagen and filaggrin levels in serum could be a useful biomarker for the diagnosis of RA.

**Table 2:** Receiver-operating characteristic curve analysis of the sensitivity, specificity, positive predictive value, negative predictive value, and accuracy for differentiating between healthy controls and rheumatoid arthritis patients according to circulating citrullinated filggrin level.

Control *vs*. RA^a^							Control *vs*. Seronegative RA						
Threshold OD	Sensitivity (%)	Specificity (%)	Likelihood ratio	PPV^b^ (%)	NPV^c^ (%)	accuracy	Sensitivity (%)	Specificity (%)	Likelihood ratio	PPV^b^ (%)	NPV^c^ (%)	accuracy
**>0.527**	**91.1**	**54.9**	**2.023**	**80.7**	**75**	**79.4**	**65**	**54.9**	**1.442**	**28.9**	**84.8**	**57.1**
**>1.013**	**78.9**	**85.9**	**5.603**	**92.0**	**66.3**	**80.8**	**40**	**85.9**	**2.84**	**47.4**	**84.7**	**76.9**
**>1.709**	**65.9**	**95.7**	**15.62**	**97.0**	**57.6**	**75.7**	**25**	**97.5**	**5.917**	**53.3**	**84.2**	**79.1**

a RA, rheumatoid arthritis; ^b^PPV, positive predictive value; ^c^NPV, negative predictive value.

### Circulating Citrullinated Antigens Are Also Detected in Seronegative RA Patients

Because the 1987 ACR criteria for RA diagnosis have low sensitivity and specificity, especially for patients with ERA (32), a new classification system for RA has been developed (22). Although the new criteria coupled with ACPA testing have improved the early diagnosis of RA, a negative result for autoantibodies (negativity for both RF and ACPA) does not rule out a diagnosis of RA. About 20% of RA patients are seronegative, meaning that no serological markers can be easily detected in the serum of these patients. Nonetheless, early diagnosis of seronegative RA is clinically important to prevent the progression of joint damage. Thus, novel diagnostic methods are needed to detect seronegative RA. In this study, the circulating level of citrullinated collagen and filaggrin was significantly higher in the sera from seronegative RA patients (n = 20) than in the controls, even though the number of samples from seronegative RA patients was small (**Figure 3C** and **Supplementary Table 1**).

Citrullinated collagen and filaggrin levels in sera of ACPA-positive RA patients were significantly higher than those of ACPA-negative RA patients or healthy controls. Interestingly, the mean OD values of both citrullinated collagen and fibronectin in the sera from ACPA-negative RA patients showed a statistically significant higher level than from healthy controls (**Figure 3D**).

These results suggest that some RA patients could have circulating citrullinated antigens but not ACPAs. Therefore, using 12G1 in an ELISA system could detect circulating citrullinated antigens and thereby aid the diagnosis of RA. Confirmation of these results in a large cohort of patients with seronegative RA is needed to determine whether this is a new method for diagnosing RF-negative, ACPA-negative RA.

### Serum Citrullinated Collagen Levels Correlate With Autoantibody Titers

We next examined the relationships between clinical characteristics and the levels of citrullinated autoantigens in RA patients to determine whether the presence of citrullinated antigens has clinical significance. The levels of none of the citrullinated antigens examined correlated with RA disease activity measured using a disease activity score of 28, erythrocyte sedimentation rate (ESR), and C-reactive protein (CRP) (data not shown). Citrullinated collagen and citrullinated filaggrin levels in sera did not differ between established RA (> 6 months duration) and ERA or young-age onset RA (onset age < 60 years) and EORA. Interestingly, the OD values for citrullinated collagen and filaggrin correlated significantly with autoantibody titers for both RF and ACPA (**Figure 3E**).

### Another Application for the 12G1 mAb: As a Mouse Model for RA

**Figure 4:**
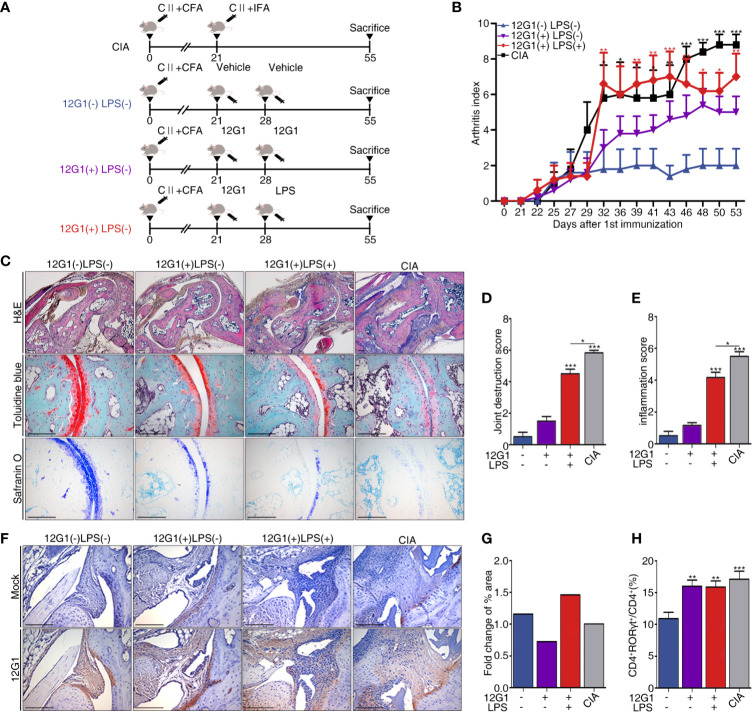
A new animal model using 12G1 mAb was constructed, and its function was confirmed by comparison with the collagen induced arthritis (CIA) model. **(A)** Schematic diagram of the CIA animal model (a standard model of rheumatoid arthritis) and the animal model using the aACPA. **(B)** Average arthritis scores for the animal models. This score is expressed on a scale of 0–4 as the sum of the arthritis severity scores for the four paws of each animal. **(C)** Histological analysis of the tarsals in the hind paws of each group stained with hematoxylin and eosin (H&E), safranin O, and toluidine blue. H&E-stained sections were magnified 50x and 100x; safranin O and toluidine blue samples are shown at 200x magnification. Histological scores were calculated as joint destruction scores **(D)** and inflammation scores **(E)** by three observers in a blind manner. **(F)** Immunohistochemical (IHC) staining of citrullinated proteins in the tarsals, magnified 100x. **(G)** Measurement of the 12G1 mAb positive cell area per whole tarsal joint area by the Slide scanner program (3D Histech Ltd, Budapest, Hungary). **(H)** Flow cytometry analysis of the CD4 and RORγt expression in the spleens of each group. The percentages of CD4+ RORγt+ divided by total CD4+ indicate the frequency of Th17 cells. All scale bars are 200µm, and all graphs are expressed using the mean ± SEM. *P < 0.05; **P < 0.01; ***P < 0.001.

We organized a new animal model using the aACPA (Materials and Methods and **Figure 4A**). We expected the 12G1 mAb to be as effective as the CIA model because previous research reported that citrullination could increase the potency of an endogenous innate immune ligand (33). CCP chelating bead was used to create a negative control in our 12G1 mAb-induced arthritis model. After binding CCP chelating beads to protein A and G of mAb 12G1’s IgG binding site, only the supernatant was inoculated into mice. We measured the arthritis score once every 3 days for 55 days after the first immunization. The score on the 55th day was the highest in the CIA group, the most commonly used standard animal model of RA, followed by the 12G1 mAb with LPS group and the 12G1 mAb only group. The vehicle-treated group showed only a minor inflammatory response in the joints (**Figure 4B**).

To analyze the infiltration of inflammatory cells, we measured the shape of cartilage and joint destruction in the tarsals using a histological analysis. In the CIA and 12G1 mAb with LPS groups, we observed more infiltration of immune cells and joint destruction than in the 12G1 mAb only group, though the 12G1 mAb with LPS group had slightly higher cartilage erosion than the other groups with the same arthritis score (**Figure 4C**). Both the inflammation score and joint destruction score were higher in the CIA and 12G1 with LPS groups than in the other groups, with small differences between them (**Figures 4D, E** **)**.

In immunohistochemical staining and immunofluorescence assays, we used the 12G1 mAb to check the amount of citrullinated protein in the joints. The 12G1 mAb with LPS group had more citrullinated protein than the CIA group (**Figure 4F**). This result was consistent when we cross-checked it with the Slide scanner viewer program (**Figure 4G**).

To compare the Th17 response, the Th17 population determined by CD4+RORγt+ expression was analyzed by flow cytometry. The results showed that Th17 cell populations were higher in the CIA and 12G1-treated groups than in the vehicle group (**Figure 4H**).

## Discussion

The artificial mAb 12G1 described herein showed high reactivity for citrullinated peptides and proteins. 12G1 detected citrullinated proteins in RA synovial tissues and was used in an ELISA to detect circulating citrullinated autoantigens in RA serum. Among the potential citrullinated autoantigen targets examined, citrullinated filaggrin showed the highest sensitivity, specificity, PPV, NPV, and diagnostic accuracy for RA, suggesting its potential as a diagnostic serological marker for RA.

Although it is not easy to detect citrullinated proteins (either whole or fragmented), previous studies have identified the presence of citrullinated autoantigens in serum, synovium, and SF from RA patients (5, 30, 34–37). Interestingly, there were two other similar attempts to detect citrulline residue of autoantigens. The first study was not designed to identify citrullinated proteins directly and chemical modification induced by incubating samples with diacetyl monoxime and antipyrine in a strong acid mixture was used to recognize citrulline (18). About 20 years ago, Nicholas and Whitaker generated a mAb, which they designated F95, that recognized citrullinated epitopes on a deca-citrulline peptide (19). By contrast, we here generated the mAb 12G1 to detect naïve citrullinated proteins directly. The ability of the 12G1 mAb to detect three potential citrullinated autoantigens (collagen, filaggrin, and fibronectin) was verified in our sandwich ELISA system.

Our mAb was generated against a synthetic peptide comprising a cyclic peptide harboring a single citrulline residue, that should allow the generation of a high-reactivity mAb. Our present study showed that the 12G1 mAb has the potential to directly detect citrullinated antigens in target tissues using immunohistochemistry. Generating a mAb by changing only a single citrulline residue (CCP versus the arginine-substituted NCP) could provide a better method for detecting naturally occurring citrullinated proteins than using the deca-citrullinated peptide. The major advantage of our mAb 12G1 is that it can detect citrullination in the sera and synovia of RA patients without prior modification of the test samples.

To investigate the concordance between the presence of circulating citrullinated autoantigens and ACPAs, we used a commercially available second-generation ACPA test to examine the sera from 115 ACPA-positive RA patients. Most of the patients whose samples exhibited mAb 12G1-reaching citrullinated proteins were also positive for ACPA (data not shown).

Interestingly, the mAb 12G1-based ELISA revealed that some ACPA-negative RA patients also had circulating citrullinated proteins. We used the 12G1 mAb-based ELISA to measure the levels of candidate proteins, including collagen, filaggrin, and fibronectin. We believe that the sensitivity and specificity of the 12G1 mAb as an RA diagnostic tool could be increased by broadening the range of target proteins and choosing different optimal cutoff values for each serological assay.

Our finding, that circulating citrullinated antigens can be detected by the 12G1-based ELISA system in RA patients at concentrations higher than those detected in healthy subjects, is consistent with a recent report implicating *in vivo* citrullination and subsequent production of ACPAs in the pathogenesis of RA (38, 39). Immunohistochemical staining detected reactivity in RA-affected synovia, whereas no positive signal was found in OA-affected or healthy synovial tissues. Our preliminary study also shows that the concentration of circulating citrullinated collagen in RA patients correlated positively with autoantibody titers (RF and ACPAs). These results suggest that the mAb 12G1 could be a useful tool in the diagnosis of RA.

## Data Availability Statement

The original contributions presented in the study are included in the article/**Supplementary Material**. Further inquiries can be directed to the corresponding authors.

## Ethics Statement

The studies involving human participants were reviewed and approved by the Institutional Review Board of Seoul St. Mary’s Hospital. The patients/participants provided their written informed consent to participate in this study. The animal study was reviewed and approved by the Institutional Animal Care and Use Committee of the School of Medicine at the Catholic University of Korea.

## Author Contributions

PW and S-JM designed the experiments. PW and YK performed the experiments and analyzed the results. S-JM performed and analyzed the results of the human samples. HJ, AR, and DS carried out the experiments and data analysis. PW, S-JM, and JJ wrote the manuscript. JJ and WR helped analyze the results. All authors read and approved the final draft of the manuscript. All authors contributed to the article and approved the submitted version.

## Funding

This work was supported by the Basic Science Research Program through the National Research Foundation of Korea (NRF), funded by the Ministry of Science, ICT, & Future Planning [grant numbers 2019R1A5A2027588, 2020R1A2C3004123], and the Institute of the Clinical Medicine Research of Bucheon St. Mary’s Hospital, Research Fund [grant number BCMC16IH01].

## Conflict of Interest

The authors declare that the research was conducted in the absence of any commercial or financial relationships that could be construed as a potential conflict of interest.
