# Supplementary material for: Pathogenic Role of Circulating Citrullinated Antigens and Anti-Cyclic Monoclonal Citrullinated Peptide Antibodies in Rheumatoid Arthritis
Source: Front Immunol. 2021 Jun 30;12:692242. doi: 10.3389/fimmu.2021.692242 (PMC8294326; doi:10.3389/fimmu.2021.692242)
Supplement: Supplementary file 1 [file DataSheet_1.docx]

**Supplementary materials**

1. **Supplementary Figure legends**

Supplemental Figure 1. Affinity of the antibodies produced by hybridoma cells for CCP

Supplemental Figure 2. Specific binding affinity and amino acid and nucleotide sequences of the mAb 12G1 antibody

1. **Supplementary Tables**

Supplemental Table 1. ELISA values for the target antigens

Supplemental Table 2. Area under the curve for each target antigen shown in Figure 3b, c

1. **Supplementary Figures**

Figure S1.

Figure S2.

**Supplementary Figure 1.** Reactivity of the antibodies produced by hybridoma cells for CCP. The ELISA was performed using supernatants from cells fused in plates coated with CCP or NCP. The cells in wells showing a positive signal against CCP were selected and cultured in new 96-well plates for a further 2 weeks. Binding reactivity for CCP was tested repeatedly. Selected clones showed a high positive signal for CCP and a negative signal for NCP compared with the negative (-) and positive (+) controls. The displayed numbers in the plate is each value of optical density (OD) of hybridoma clones. Grey boxes show clones specific for CCP.

**Supplementary Figure 2.** Amino acid and nucleotide sequences of the mAb 12G1 antibody. (a) specific binding affinity of mAb 12G1 to CCP. (b) The light chain and heavy chain sequences are shown. The complementarity-determining regions (CDRs) are underlined.

**Supplementary Table 1.** ELISA values for the collagen and filaggrin

| Target antigens | **ELISA result (mean OD ± SD)** | | ***P* value^a^** | **ELISA result (mean OD ± SD)** | ***P* value^b^** |
| --- | --- | --- | --- | --- | --- |
|  | HC | RA |  | Seronegative RA |  |
|  | (n = 71) | (n = 148) |  | (n = 20) |  |
| Citrullinated collagen | 0.381 ± 0.531 | 1.553 ± 1.103 | <0.0001 | 0.828 ± 0.709 | < 0.05 |
| Citrullinated filaggrin | 0.648 ± 0.408 | 2.226 ± 1.071 | <0.0001 | 1.208 ± 0.911 | < 0.05 |

^a^: HC versus RA, ^b^: HC versus seronegative RA

**Supplementary Table 2.** Area under the curve for each target antigen shown in Figure 4

| **Target antigens** | **Whole RA** | | |  | **Seronegative RA** | | |
| --- | --- | --- | --- | --- | --- | --- | --- |
|  | **AUC** | **95% CI**^a^ | ***P* value** |  | **AUC** | **95% CI** | ***P* value** |
| Citrullinated collagen | 0.817 | 0.761-0.874 | <0.0001 |  | 0.645 | 0.528-0.762 | <0.05 |
| Citrullinated filaggrin | 0.891 | 0.849-0.933 | <0.0001 |  | 0.681 | 0..530-0.832 | <0.05 |

^a^CI, confidence interval.

**Supplementary fig.1**

**
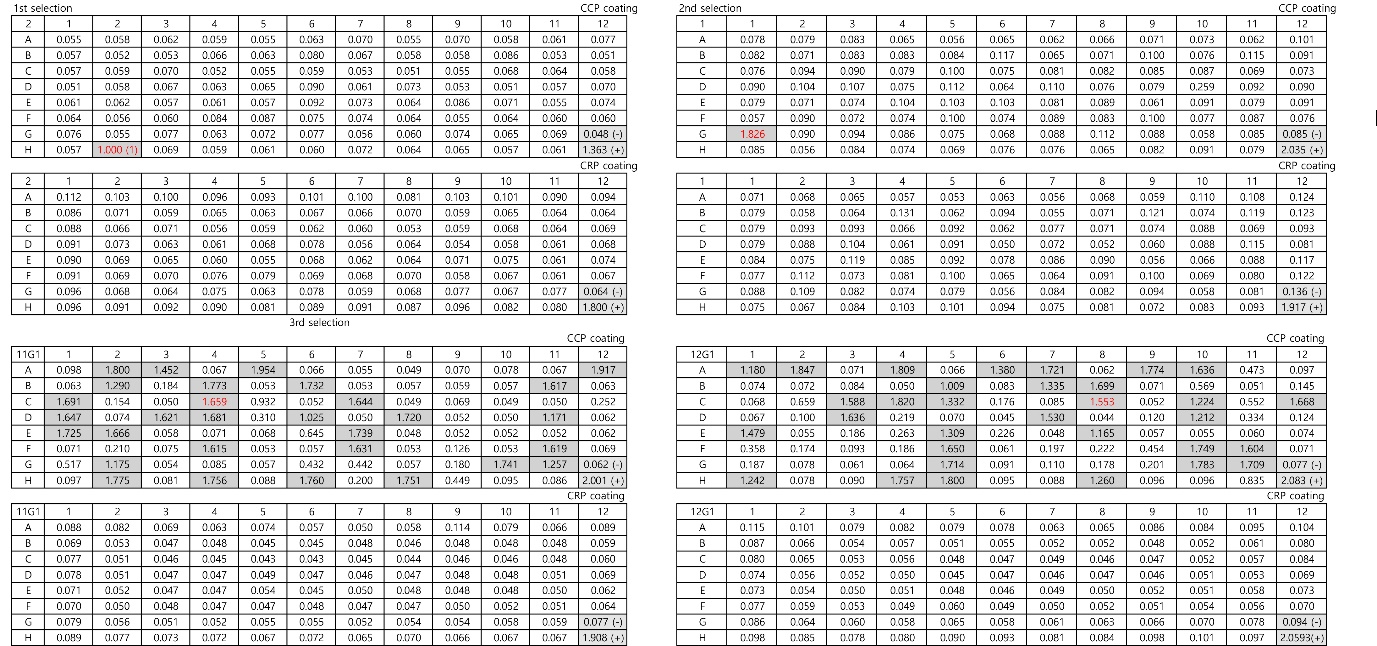
**

**Supplementary fig.2**

**
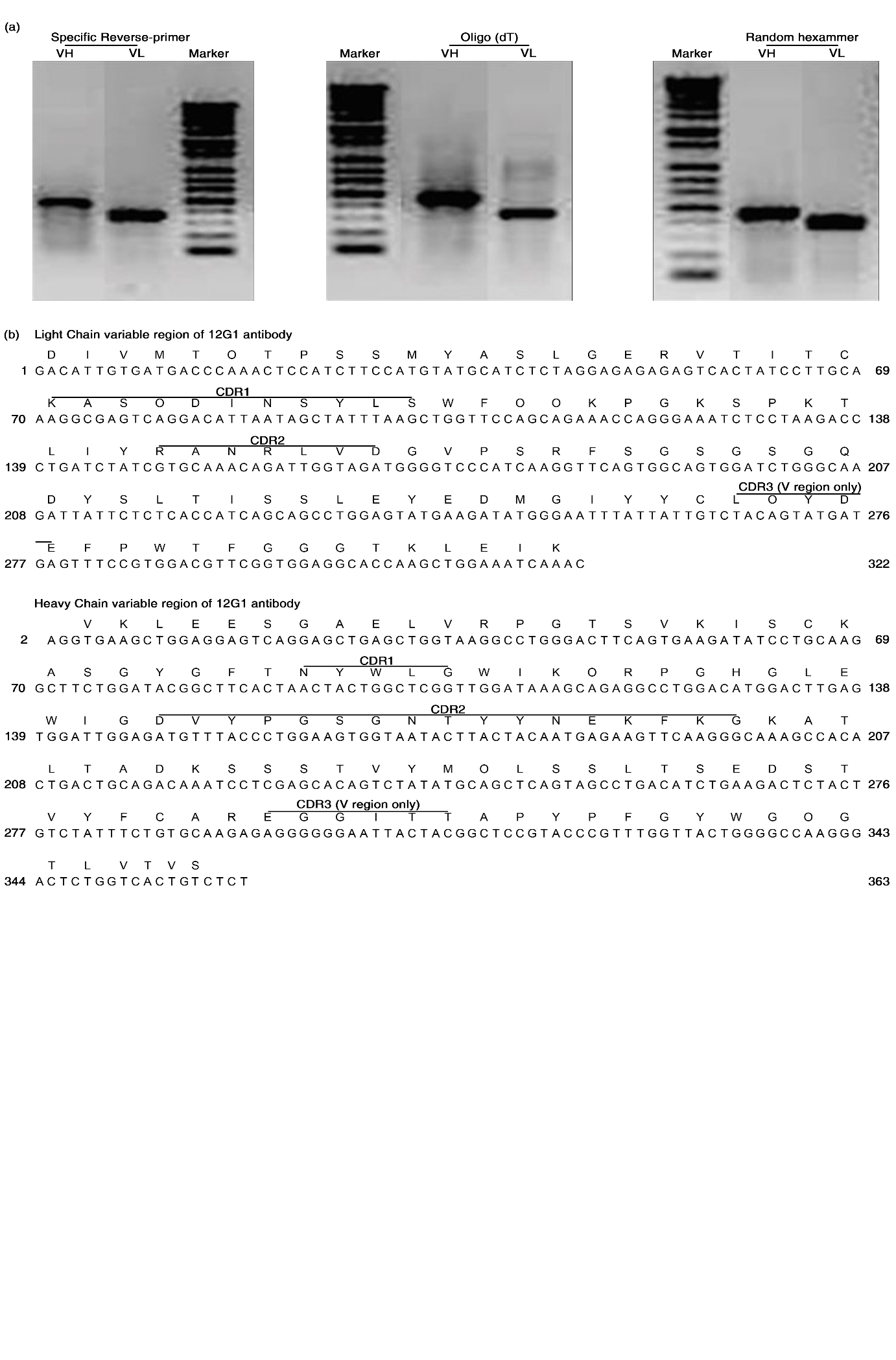
**
